# An interpretable data-driven approach to optimizing clinical fall risk assessment

**DOI:** 10.1371/journal.pdig.0001631

**Published:** 2026-09-01

**Authors:** Fardin Ganjkhanloo, Emmett Springer, Erik H. Hoyer, Daniel L. Young, Holley Farley, Kimia Ghobadi

**Affiliations:** 1 Center for Health Systems and Policy Modeling, Department of Health Policy and Management, Johns Hopkins University, Baltimore, Maryland, United States of America; 2 Malone Center for Engineering in Healthcare, Johns Hopkins University, Baltimore, Maryland, United States of America; 3 Center for Systems Science and Engineering, Department of Civil and Systems Engineering, Johns Hopkins University, Baltimore, Maryland, United States of America; 4 Department of Physical Medicine and Rehabilitation, School of Medicine, Johns Hopkins University, Baltimore, Maryland, United States of America; 5 Department of Physical Therapy, University of Nevada, Las Vegas, Las Vegas, Nevada, United States of America; 6 Department of Nursing, The Johns Hopkins Hospital, Baltimore, Maryland, United States of America; Shanghai United Imaging Intelligence Co Ltd, CHINA

## Abstract

In this study, we aim to better align fall risk prediction from the Johns Hopkins Fall Risk Assessment Tool (JHFRAT) with clinical expert fall risk perception via a data-driven modelling approach. We conducted a retrospective cohort analysis of 54,209 inpatient admissions from three Johns Hopkins Health System hospitals between March 2022 and October 2023. In the absence of a true fall risk ground truth, we apply proxy labels based on clinician choices for the application of targeted preventative interventions, resulting in a total of 20,208 high-risk encounters and 13,941 low-risk encounters. We employed constrained score optimization (CSO) models to recalibrate the JHFRAT scoring weights, while preserving its additive structure and clinical thresholds. Recalibration refers to adjusting item weights so that the resulting score can order encounters more consistently by the study’s risk labels, and without changing the tool’s form factor or deployment workflow. The CSO model demonstrated significant improvements over the current JHFRAT in classification alignment with the proxy labels (CSO AUC-ROC = 0.91, JHFRAT AUC-ROC = 0.86). This model performance translates to a weekly average of an additional 35 Johns Hopkins Health System patients who are perceived as high risk (per our proxy labels) being classified by JHFRAT as high risk. The ablation analyses also suggest that the CSO model, though outperformed in prediction metrics by the benchmark black-box XGBoost model, is more robust than XGBoost to variations in risk labeling. Our evidence-based approach provides a robust foundation for understanding risk factor contributions to various indicators of clinician-perceived fall risk. Future research can build upon this foundation to improve risk assessment utility as a decision-support tool in clinical practice.

## Author summary

Inpatient falls are a major safety concern in hospitals, often leading to injuries and longer recovery times. To prevent these events, nurses use standardized tools like the Johns Hopkins Fall Risk Assessment Tool to identify vulnerable patients and provide extra care, such as more frequent check-ins or specialized equipment. However, these tools are often based on general clinical expertise rather than precise data, and their accuracy can be hidden because the very interventions they trigger prevent the falls they are trying to predict. We addressed this challenge by analyzing over 54,000 hospital admissions using a data-driven approach that accounts for the preventative care patients were assigned by nurses. By recalibrating the original assessment tool with advanced mathematical modeling, we significantly improved its alignment with clinician intuition of fall risk patients without making the tool more difficult for nurses to use. Analyzing fall risk assessment in this manner is a crucial first step to improving and standardizing clinical practice to simultaneously promote patient mobility and reduce the number of preventable falls.

## 1. Introduction

Inpatient falls remain a critical issue within hospital settings, leading to increased morbidity, mortality, and significant healthcare costs [1]. Fall-related injuries, particularly among older adults, result in prolonged hospital stays, reduced quality of life, and substantial resource demands on healthcare systems [2,3]. More broadly, falls represent a prototypical hospital-acquired harm for which prevention depends on accurate risk stratification, timely clinical decision-making, and judicious allocation of limited resources. Widespread implementation of intensive risk-mitigation interventions, such as increased rounding and restriction of patient mobility, is resource-intensive and may offer limited value for patients with low fall risk [4,5]. At the same time, underestimating or failing to identify patients truly at high risk can result in missed opportunities for timely intervention and prevention of serious harm. Reliable fall risk assessment is essential for implementing targeted prevention strategies and optimizing resource allocation, a challenge shared across many hospital safety domains where adverse events are uncommon, but consequences are substantial.

However, developing reliable models of fall risk directly from observed fall outcomes is fundamentally challenging for several reasons. Falls are relatively rare events, occurring in fewer than 1% of inpatient encounters, making it statistically difficult to learn meaningful risk signal from outcome data alone. Even among genuinely high-risk patients, falls retain an inherent stochastic component, such that a patient may be at elevated risk during a given shift and not fall simply by chance. Furthermore, fall prevention interventions are often applied preemptively to those perceived as high risk, so that patients who receive effective interventions are less likely to fall despite their elevated underlying risk. The resulting data often reflect patterns of clinical decision-making as much as true fall likelihood, systematically obscuring the relationship between patient risk factors and observed outcomes. Finally, documentation of fall events is subject to inconsistency, as unwitnessed falls and near-misses may not be uniformly captured, further reducing the reliability of outcome-based labels.

Given these challenges, we propose that clinician-perceived fall risk, as expressed through consistent nurse decision-making about targeted fall prevention interventions, represents a more accessible and well-defined target for data-driven risk modeling. While clinician-perceived risk cannot be observed directly, it can be operationalized through intervention patterns: a nurse who consistently applies several targeted interventions throughout an encounter is implicitly signaling a high-risk perception, and one who applies very few is signaling low risk. This operationalization is well-motivated because the JHFRAT itself is designed to guide exactly these intervention decisions. Recalibrating it to better align with empirical decisions for the purposes of standardizing care best practices is therefore a clinically coherent goal.

The Johns Hopkins Fall Risk Assessment Tool (JHFRAT) [6] is widely used in hospitals to score fall risk based on seven domains assessed by nursing staff once per shift and as patient condition changes. However, its scoring system and risk thresholds were developed using an evidence-based approach [7], not empirically derived, raising concerns about their ability to represent the complex, dynamic nature of fall risk. Furthermore, the reliance on clinical judgement and absence of an empirical ground truth for an individual’s true fall risk complicates validation and model refinement. While some previous research utilizes “off-the-shelf” machine learning models to predict inpatient fall risk [8–12], few address the risk-obscuring effects of interventions [13] or seek to optimize existing clinical assessments with a focus on interpretability and assuredness, features that are critical for decision support tools intended for frontline use across hospital safety domains.

In this study, we use a “score-based recalibration” by adjusting the relative weights assigned to JHFRAT items (and their implied category separation) so that higher total scores correspond to higher risk labels more consistently. Our goal is to improve the ranking and category assignment of an existing clinical score, while preserving its interpretability and clinical use pattern by (1) assigning proxy labels for clinician perceived fall risk based on observed fall prevention practices; (2) applying a constrained score optimization framework to recalibrate JHFRAT coefficients and incorporate additional electronic health record (EHR) clinical variables; and (3) evaluating the sensitivity of the optimized model outputs to clinically informed proxy label selection rules, including explicit incorporation of NDNQI-adjudicated fall occurrences. Through this multi-site analysis, we aim to determine whether data-driven modeling can enhance the calibration of an established risk assessment tool to clinical expertise without compromising its transparency or ease of implementation. The methods presented have broad applicability to risk assessment in other healthcare contexts where adverse events are rare or ground-truth risk is greatly obscured by intervention. Although falls serve as the motivating use case, the methods presented provide a proof of concept for improving risk assessment in other hospital-acquired harms characterized by rare outcomes, preventive interventions, and decision-dependent data generation.

## 2. Materials and methods

### 2.1. Data source

We follow the TRIPOD and STROBE statement guidelines for reporting the study data source and methodology [14]. Data for this study were extracted from electronic health records for all adult inpatient encounters (excluding psychiatric, labor and delivery, and observation units) with admission and discharge between March 28^th^ 2022 and November 3^rd^ 2023 across three Johns Hopkins Health System hospitals: Johns Hopkins Hospital (JHH), Johns Hopkins Bayview Medical Center (BMC), and Howard County Medical Center (HCM). A total of 54,209 encounters from the selected period had lengths of stay (LOS) of at least 48 hours and constitute the study data set. The Johns Hopkins Fall Risk Assessment Tool (JHFRAT) consists of 18 binary risk variables across seven categories: age, cognition, elimination, fall history, patient care equipment, medication, and mobility. Each risk variable has an associated point value (Table 4, Column 'Baseline: JHFRAT'), which adds to a total risk score. Patients are classified by their total JHFRAT score as low-risk (<6), moderate-risk (6–13) or high-risk (>13). In addition to the JHFRAT risk variables, nurses are required to record what fall prevention interventions, if any, the patient will receive (or is currently receiving). There are 30 standardized intervention options that can be recorded as binary variables, and we retain the records for these variables in the data source along with each recorded JHFRAT.

**Table 1:** Risk labels across sensitivity analysis cohorts. While the low-risk-labeled population remains stable, the high-risk-labeled population shrinks as the high-intervention window threshold is increased.

High-Intervention Window Threshold	Number of Encounters per Risk Label		
	Low	High	Indeterminate
**4**	19,766	20,368	6,908
**5**	20,077	16,675	10,290
**6 (Main Cohort)**	20,269	13,837	12,936
**7**	20,394	11,363	15,285
**8**	20,458	9,443	17,141

The extracted EHR data also includes daily measures of patient mobility with the Johns Hopkins Highest Level of Mobility (JH-HLM) [15] scores, Activity Measure for Post-Acute Care Basic Mobility ‘6-clicks’ Short Form (AM-PAC) [16] scores, age, gender, race, service category, and number of documented comorbidities. These additional variables were selected for inclusion in the data source based on established clinical relevance [17–20] to fall risk assessment and data availability within the EHR system. The JHFRAT, AM-PAC, and JH-HLM are mandated to be completed once during each 12-hour nursing shift, and the study data include all completed assessments for each encounter. Demographic variables, the encounter department service category, and comorbidity counts are documented once per encounter in the data source.

This health system follows the National Database for Nursing Quality Indicators (NDNQI) guidelines for fall and fall with injury data collection and reporting. During the study period, 498 encounters (0.92% of encounters) included at least one fall event documented and later reviewed in accordance with the NDNQI guidelines. There are 623 total documented falls among these encounters, resulting in 1.5 falls per 1,000 patient-days across all encounters in the data. We received approval for the study protocol from the Johns Hopkins Institutional Review Board, and data collection followed established privacy protection guidelines.

### 2.2. Study design

The primary target of this study is clinician-perceived fall risk, defined as the encounter-level clinical judgment of a patient’s fall risk as expressed through the consistent application of targeted fall prevention interventions. Direct observation of this construct is not feasible at the scale of our dataset, as it would require individual case reviews by clinical experts for each encounter. We therefore operationalize clinician-perceived fall risk through an intervention-based proxy labeling framework, on the premise that a nurse who consistently applies several targeted interventions throughout an encounter is expressing a high-risk clinical judgment, while one who consistently applies very few is expressing a low-risk judgment. The following describes the cohort construction and labeling procedure used to implement this framework.

Any encounters with fewer than three completed JHFRAT records in the data are excluded. To ensure sufficient longitudinally of analyzed data, we exclude encounters with a length of stay fewer than two full days and exclude any patients whose first fall was before the 3^rd^ full day of the encounter. Furthermore, we exclude patients with a length of stay greater than 21 full days to avoid introducing clinical outliers to the analysis set. Counts and characteristics of the excluded encounters are included in Section 3.1.

We conducted structured clinical review sessions with clinical experts from nurse quality and physical medicine and rehabilitation to characterize interventions among the 30 options as standard or targeted, based on clinical expertise. A targeted intervention is defined for the purpose of this study as a resource-intensive measure not suitable for broad implementation but rather reserved for only high fall risk patients. The following 12 interventions were identified as targeted: increased rounding, injury prevention hospital protocol (e.g., bleeding risk, fracture risk), PT/OT consult, frequent reorientation, bed/chair exit alarm, bladder management device, elimination schedule, fall prevention education to caregivers, early delirium recognition and treatment, physical bed restraints, constant observation, and protective devices (e.g., hip protector, floor mat, helmet, low bed). Interventions considered standard include ensuring patient is safe and independent with use of assistive device, keeping equipment on one side of patient, and educating patient to call/wait prior to mobilizing.

**Fig 1:**
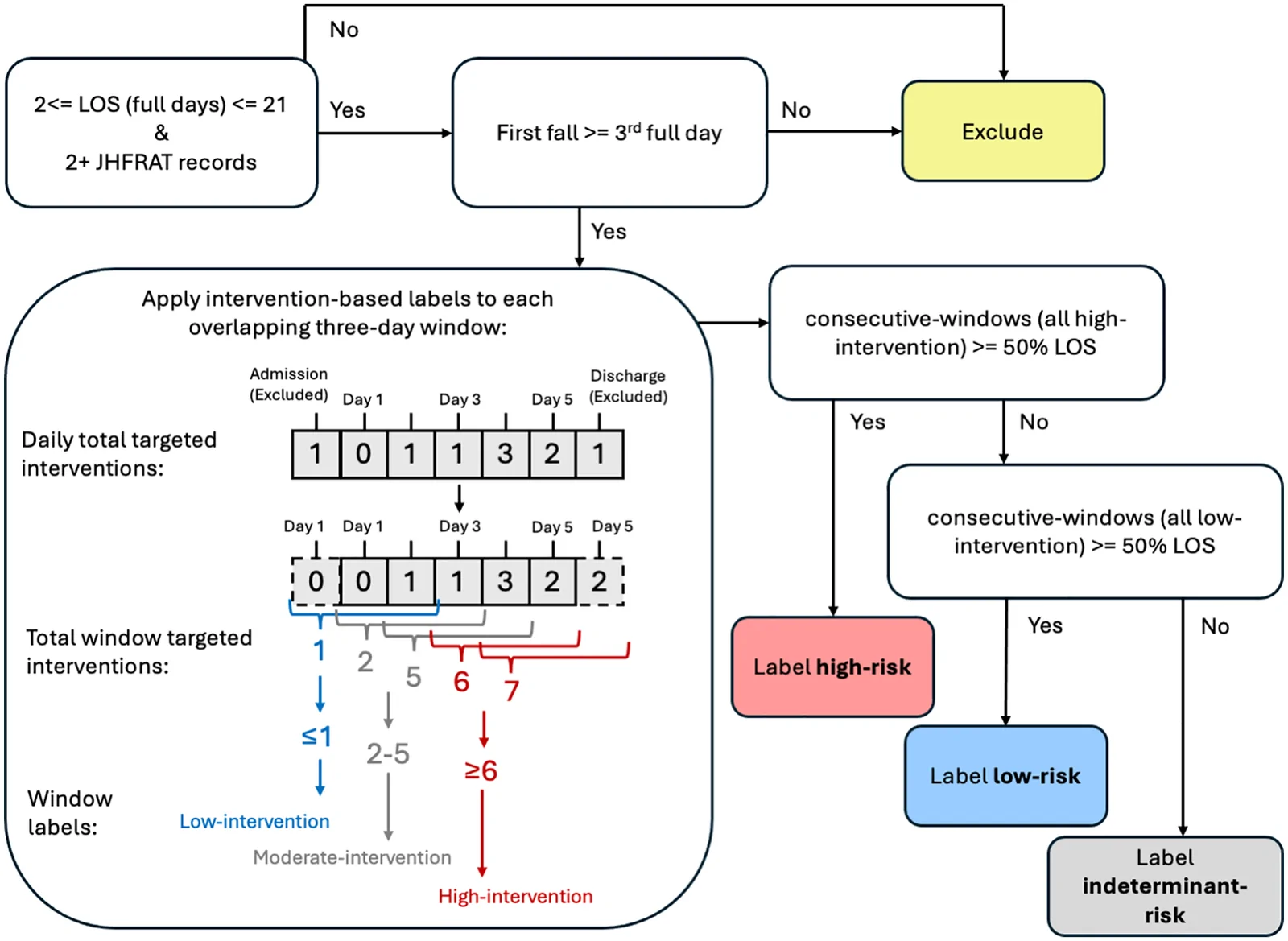
Encounter filtering and time-smoothed intervention-based proxy labeling algorithm. The low-intervention window threshold is 1, the high-intervention window threshold is 6, and the moderate-intervention range is 2-5 total targeted interventions over the three-day window. To be labeled as high-risk or low-risk, an encounter must have a stretch of consecutive windows which span at least half of the full-day length of stay, which are labeled all high-risk or low-risk, respectively.

Our primary approach for data labeling is a per-encounter, time-smoothed, targeted intervention-based proxy label, which aims to capture expert perception of patient fall risk. In lieu of conducting case reviews for all encounters (a monumental task for the size of our dataset), we quantify the consistent application (or lack thereof) of targeted fall prevention interventions. These labels represent the encounter-average clinician perception of fall risk since they are derived from the clinician decisions on the intensity of appropriate prevention intervention. To smooth over small daily variabilities in clinical decision-making, we consider all overlapping time windows of three consecutive full calendar days. We adjusted for the beginning and end of the encounter by considering the first and last day twice, per the algorithmic illustration in Fig 1. With the addition of these days, each actual day is included in exactly three three-day windows, which avoids biasing the encounter labels towards the days in the middle of the encounter. As illustrated in Fig 1, we label each three-day window as either low-intervention, moderate-intervention, or high-intervention depending on the total number of targeted interventions applied over the three days of the window. Then, we apply intervention-based proxy labels to each encounter (either low-risk, high-risk, or indeterminate-risk) according to the decision tree in Fig 1. For example, an encounter with a LOS of six full days would have six three-day windows. A consecutive stretch of three of these windows must be either low-intervention or high-intervention to be labeled as low-risk or high-risk respectively. We exclude the indeterminate-risk labeled patients from model training and evaluation, allowing the use of binary classification methods and metrics.

In addition to the main cohort, derived from the intervention-based proxy label criteria above, we conduct the following three ablation analyses on the cohort composition, with a focus on the criteria for applying the high-risk label.

*Fall occurrence high-risk override:* Utilizing the same labels for all non-fall encounters as the main cohort, we apply high-risk labels to all fall encounters from the main cohort, regardless of prevention interventions. The goal of this analysis is to assess the difference in optimal JHFRAT model parameters when the emphasis in training is placed on the occurrence of NDNQI-adjudicated falls rather than the clinician perception proxy labels. To accomplish this, we apply additional weight in the objective to the high-risk labeled training samples that are fall encounters and perform sensitivity on this hyperparameter.

*High-intervention window threshold:* Utilizing the same intervention-based proxy labeling approach as above, we vary the high-intervention window label threshold between four and eight. The resulting cohorts vary in the conservativeness of the high-risk label as it relates to inferred clinician perspectives of risk via intervention application.

*Prior-to-falls intervention pattern matching:* Utilizing the main cohort labels as a starting point, we apply a prior-to-falls intervention pattern matching procedure to the main cohort by matching fall encounters labeled as high risk in the main cohort to encounters labeled as indeterminate (fall and non-fall) in the main cohort. For each of these high-risk fall encounter three-day windows, we identify up to three indeterminate encounters that have a stretch of three days with the exact same configuration (all applied interventions alike, no interventions that are not present in the high-risk fall encounter window) of targeted interventions. Ties are broken for the candidate-matching encounters first by the alignment (per L1 norm of the two binary intervention vectors) of non-targeted interventions across the matching three-day window, then by how closely the windows align temporally to the starts of their respective encounters (i.e., a match of days 4–6 is closer to the high-risk fall window of days 3–5 than a match of days 7–9). Remaining ties are broken by closeness of the hospital admission time of the candidate match to the hospital admission time of the high-risk faller.

After following this matching procedure, similarly to the falls-based weighting in the analysis described above, we apply an additional weight in the objective to the high-risk labeled patients and their intervention-based matches. The purpose of this analysis is to assess the model response to tuning the proxy labels to the specific patterns of frequent interventions applied prior to falls rather than the general consistency of targeted intervention implementation captured by the main cohort time-smoothed targeted intervention implementation labeling approach.

**Fig 2:**
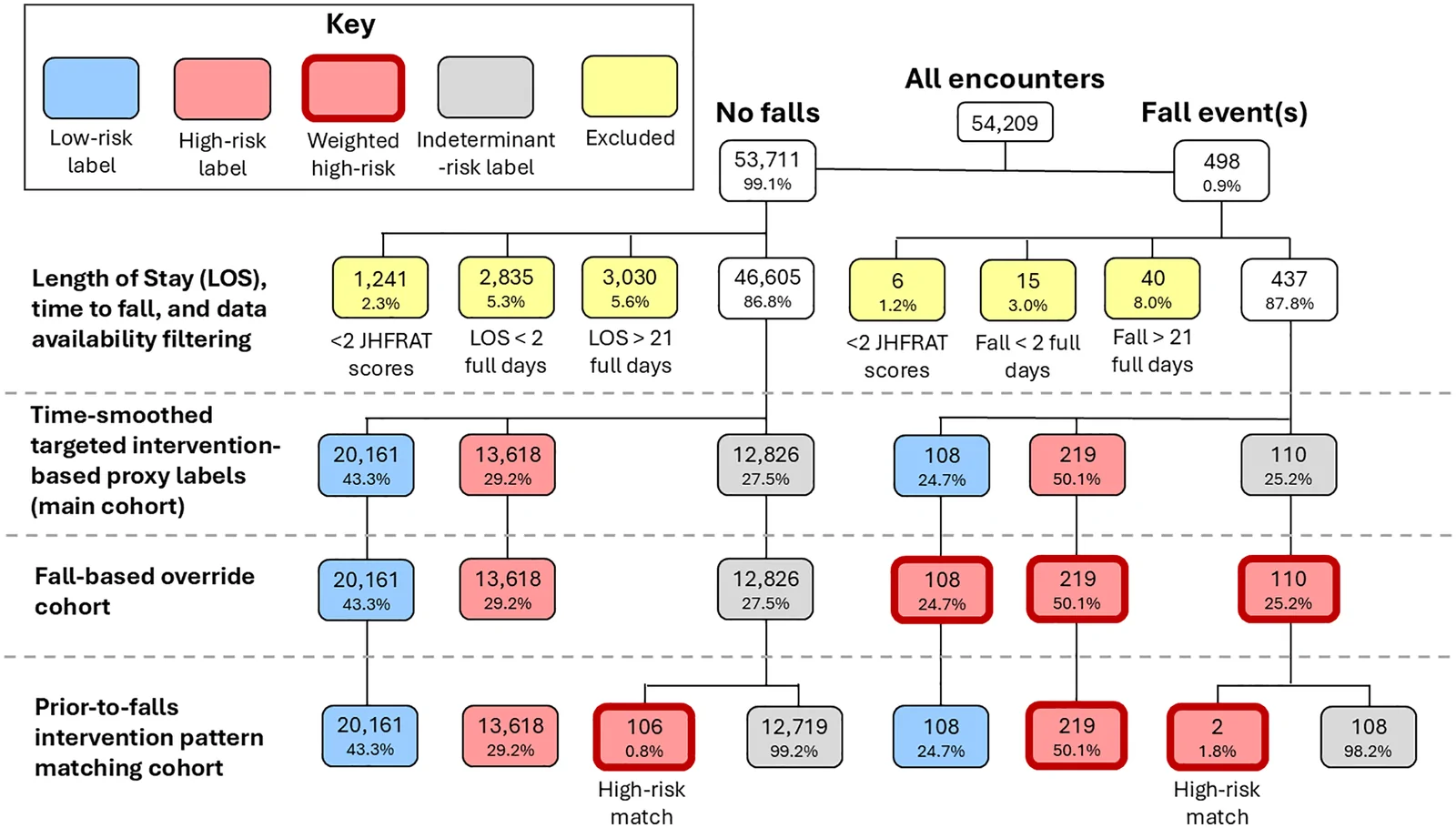
Encounter exclusion and cohort selection steps and outcomes. The final labeled study cohort (low-risk and high-risk labels only) comprises 63.1% of the 54,209 encounters in the initial data set. “High-risk match” encounters in the prior-to-falls intervention pattern matching cohort are obtained by exact matching on the targeted intervention profile of any three-day window of an indeterminate encounter to the three-day window targeted intervention profile of any of the 219 fall encounters labeled high-risk in the main cohort.

The full cohort filtering and labeling cohort composition results are illustrated in Fig 2. For all cohort variations, we perform stratified 5-fold cross-validation with samples assigned to folds prior to model training such that all models for a given cohort are trained and evaluated on the same folds. Folds are stratified by risk-label for all cohort variations and are further stratified by fall status and matching status (high-risk via main cohort vs high-risk via matching) for the relevant ablation analyses. Samples labeled as high-risk via matching are assigned to the same folds as the high-risk fall sample to which they were matched to avoid data leakage. Sensitivity to labeling approach is evaluated by examining the feature importance variation between the models trained on the different cohorts and weighting hyperparameters.

### 2.3. Model implementation

We employ two complementary modeling approaches to both quantify the performance gains achievable through additional EHR data and compare the performance of the white-box and black-box models for alignment of samples with proxy labels. First, we employed a JHFRAT-specific implementation of the recently developed generalized constrained score optimization (CSO) model for ordinal classification [21] that preserves the structure of the JHFRAT assessment tool by modifying the individual risk item score coefficients. Let $X\in R^{n\times m}$ represent the feature matrix for n training samples and m risk factors, and $y\in \{\text{0},\text{1}{\}}^{n}$ denote the binary risk label (low-risk = 0, high-risk = 1). Encounter-specific objective weights are defined by $w_{i}$. Let C represent the set of ordered pairs (j,k) encoding clinical hierarchies in scores. The optimization problem seeks to determine the set of risk variable coefficients $\beta \in R^{\text{m}}$ that maximize correct classification of encounter samples to their proxy labels while satisfying clinical constraints via the following formulation:


$$\begin{array}{cccc} \text{CSO}(X,y,\lambda ):\; & \underset{\beta }{\max}\;\lambda L_{T_{\text{low}}}+(1-\lambda )L_{T_{\text{high}}}\\ \text{s.t.}\; & L_{T}=\sum_{i=1}^{n}w_{i}(y_{i}(X_{i}\beta -T)-\ln(1+e^{X_{i}\beta -T}))\; & & \forall \;T\in \{T_{\text{low}}=6,\;T_{\text{high}}=13\}\\ & \beta_{j}\leq \beta_{k}\; & & \forall \;(j,k)\in C\\ & \beta_{j}\geq 0\; & & \forall \;j\in [m],\\ \text{where}\; & w_{i}=\left\{ \begin{array}{cc} 1/(1-\sum_{i=1}^{n}y_{i}) & \text{if }y_{i}=0,\\ 1/(\sum_{i=1}^{n}y_{i}) & \text{if }y_{i}=1 \end{array}\right. \end{array}$$

The risk score for each encounter is defined by $X_{i}\beta$, maintaining the additive structure of JHFRAT. The optimization objective is a weighted combination of the score log-likelihoods, $L_{T}$, with two different thresholds: $T_{low}=\text{6}$ and $T_{high}=\text{1}\text{3}$. These thresholds match the current JHFRAT category thresholds between the low-risk and moderate-risk categories ($T_{low}=\text{6}$) and between the moderate-risk and high-risk categories ($T_{high}=\text{1}\text{3}$). Each $L_{T}$ objective term is the logistic regression objective for a different binary logistic regression task, with the parameter λ controlling the relative importance of the two regression objectives. The $L_{T_{low}}$ objective promotes classifying samples labeled low-risk in the JHFRAT low or moderate-risk categories and classifying samples labeled high-risk as only high-risk, while the $L_{T_{high}}$ objective promotes classifying samples labeled low-risk only as low risk and high-risk labeled samples as either moderate or high-risk. We run all experiments with λ=0.5.

The weighted sample normalization via $w_{i}$ helps address the class imbalance in our dataset by making the total weight per class equal in the objective. In the primary analysis and high-risk total intervention threshold ablation analysis, the formula given above for ${\text{w}}_{\text{i}}$ is used. The falls-based and matching-based ablation analyses incorporate an additional hyperparameter, α, which is the relative weight given to a sample in the group of interest (fallers or matched samples, indicated by $y_{i}*=\text{1}$) relative to the high-risk labeled samples not in the group of interest (indicated by $y_{i}*=\text{0}$). The following formulation for $w_{i}$ is applied for these two analyses to maintain overall equal weight per class in the CSO objective while testing model sensitivity to favoring correct classification for the groups of interest.


$$\begin{array}{c} w_{i}=\left\{ \begin{array}{cc} 1/(1-\sum_{i=1}^{n}y_{i}) & \text{if }y_{i}=0,\\ 1/(\sum_{i=1}^{n}y_{i}(1-y_{i^{*}})+\alpha \sum_{i=1}^{n}y_{i^{*}}) & \text{if }y_{i}=1,y_{i^{*}}=0\\ \alpha /(\sum_{i=1}^{n}y_{i}(1-y_{i^{*}})+\alpha \sum_{i=1}^{n}y_{i^{*}}) & \text{if }y_{i}=1,y_{i^{*}}=1 \end{array}\right. \end{array}$$

The coefficient ordering constraints incorporate structured clinical knowledge by preserving the ordinal relationships within each of the following single-select JHFRAT categories: Age, Medications, and Patient Care Equipment. We therefore ensure that higher levels of assessed risk factors contribute to progressively greater risk scores than lower levels within the same domain. Non-negativity constraints guarantee positive risk contributions. Notably, this optimization architecture readily accommodates additional constraint formulations, enabling systematic incorporation of evolving clinical knowledge or institution-specific requirements.

We train two versions of the CSO models: Optimized CSO, which utilizes only the 18 current JHFRAT risk factors as features, and Augmented CSO, which includes 22 additional binary EHR variables (Table 4), that include demographics, AM-PAC and JH-HLM score ranges, and department service category. The CSO model is trained on both the JHFRAT-only and expanded datasets to establish empirical performance benchmarks achievable through unconstrained utilization of the expanded variable set. Since each of the JHFRAT assessment components and the JH-HLM and AM-PAC scores are documented multiple times throughout each encounter, we utilize their average value across the encounter for model training and evaluation and do not impute any values for assessments missed in a shift. All CSO models are solved with the CVXPY optimization library MOSEK solver with a convergence threshold of 10^-8^ and 10^6^ maximum iterations.

We additionally train common benchmark models with the best performance achieved by gradient boosted decision tree models (XGBoost). All XGBoost models are trained using the Python XGBoost library with hyperparameters (100 estimators, learning rate of 0.1, no maximum depth) selected through preliminary exploration. Since the XGBoost model does not assign each data sample a risk score, we create a projected risk score from the XGBoost output to compute all reported binary performance metrics (F1, sensitivity, specificity, true positive rate, false positive rate, precision, and recall). The projected risk score is the model output probability of belonging to the high-risk label class multiplied by the maximum possible current JHFRAT score, 35.

## 3. Results

### 3.1. Study cohort composition

A total of 46,605 non-fall encounters and 437 fall encounters meet the length of stay, time to fall, and JHFRAT data availability criteria for study cohort inclusion, as illustrated in Fig 2. Demographics and daily occurrence rates of all model variables for both the excluded and included encounters are detailed in Section 3.3. Applying the time-smoothed targeted intervention-based proxy labels results in a main study cohort of 34,101 training samples, with 20,265 (59.4%) encounters labeled low-risk, 13,836 (40.6%) as high-risk, and 12,935 labeled as indeterminate-risk and thus excluded from training. The fall and non-fall groups have similar rates of indeterminate labeled encounters, but a higher portion of fall encounters (50.1%) are labeled as high-risk compared to the non-fall encounters labeled high-risk (29.2%). The patient-day normalized rate of falls for high-risk labeled patients is double that of low-risk patients, with 1.90 falls per 1,000 patient days, compared to 0.89 falls per 1,000 patient days for the low-risk labeled cohort. Meanwhile the indeterminate-labeled encounters have 1.50 falls per 1,000 patient days.

**Table 2:** Average performance metrics across 5-fold cross-validation for binary classification across original JHFRAT and four trained models.

Metric	Threshold	Original JHFRAT	Optimized CSO	Augmented CSO	Optimized XGBoost	Augmented XGBoost
F1	6	0.72	0.76	0.76	0.78	0.80
	13	0.43	0.67	0.69	0.84	0.86
Sensitivity	6	0.95	0.96	0.97	0.97	0.97
	13	0.29	0.54	0.57	0.91	0.93
Specificity	6	0.54	0.61	0.61	0.65	0.69
	13	0.96	0.95	0.95	0.82	0.84
AUROC	N/A	0.86	0.90	0.91	0.95	0.96
AUPRC		0.77	0.84	0.85	0.93	0.94

Table 1 provides the number of encounters per intervention-based proxy label for the sensitivity analysis on the high-intervention window threshold. The fall-based cohort overrides the low-risk and indeterminate risk labels on 108 and 110 fall encounters, respectively, to label all fall encounters as high-risk. The prior-to-falls intervention pattern matching identifies 45 of the high-risk fall encounters with at least one indeterminate encounter match, yielding a total of 108 indeterminate encounters re-labeled as high-risk via matching.

**Fig 3:**
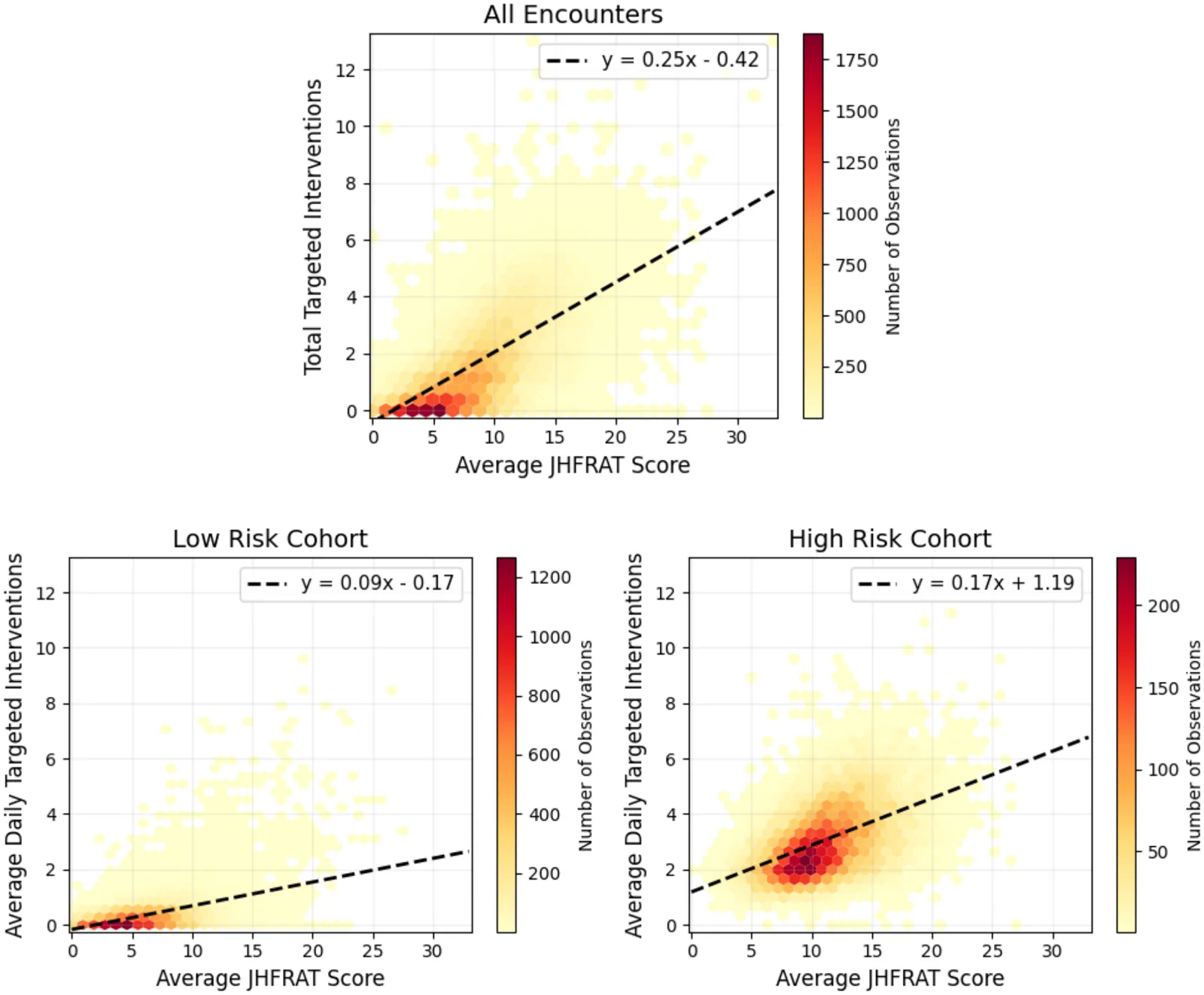
Correlation between encounter average JHFRAT scores and average daily number of targeted interventions for all encounters (above), encounters in the low-risk study cohort (below left, 20,265 encounters) and encounters in the high-risk study cohort (below right, 13,836 encounters). JHFRAT score and average daily targeted interventions are correlated, with both generally higher in the high-risk cohort.

The Spearman correlation between average daily JHFRAT score and total daily targeted interventions for all encounters in the main study cohort is 0.61 (p < 0.001), indicating that patients with higher JHFRAT scores typically receive more targeted interventions overall, as intended by the intervention-based proxy label approach. However, Fig 3 demonstrates how the number of interventions varies widely between patients with similar average daily JHFRAT scores despite the clear correlation. Across all encounters, the scores are largely clustered below 10, with fewer than 2 targeted interventions per day on average. When separated by risk label, there is a notable difference in the data distributions. The low-risk group is even more highly concentrated towards few daily targeted interventions, while the high-risk group’s data clusters between 2 and 6 daily targeted interventions and JHFRAT scores between 5 and 15.

### 3.2. Model performance comparison

**Fig 4:**
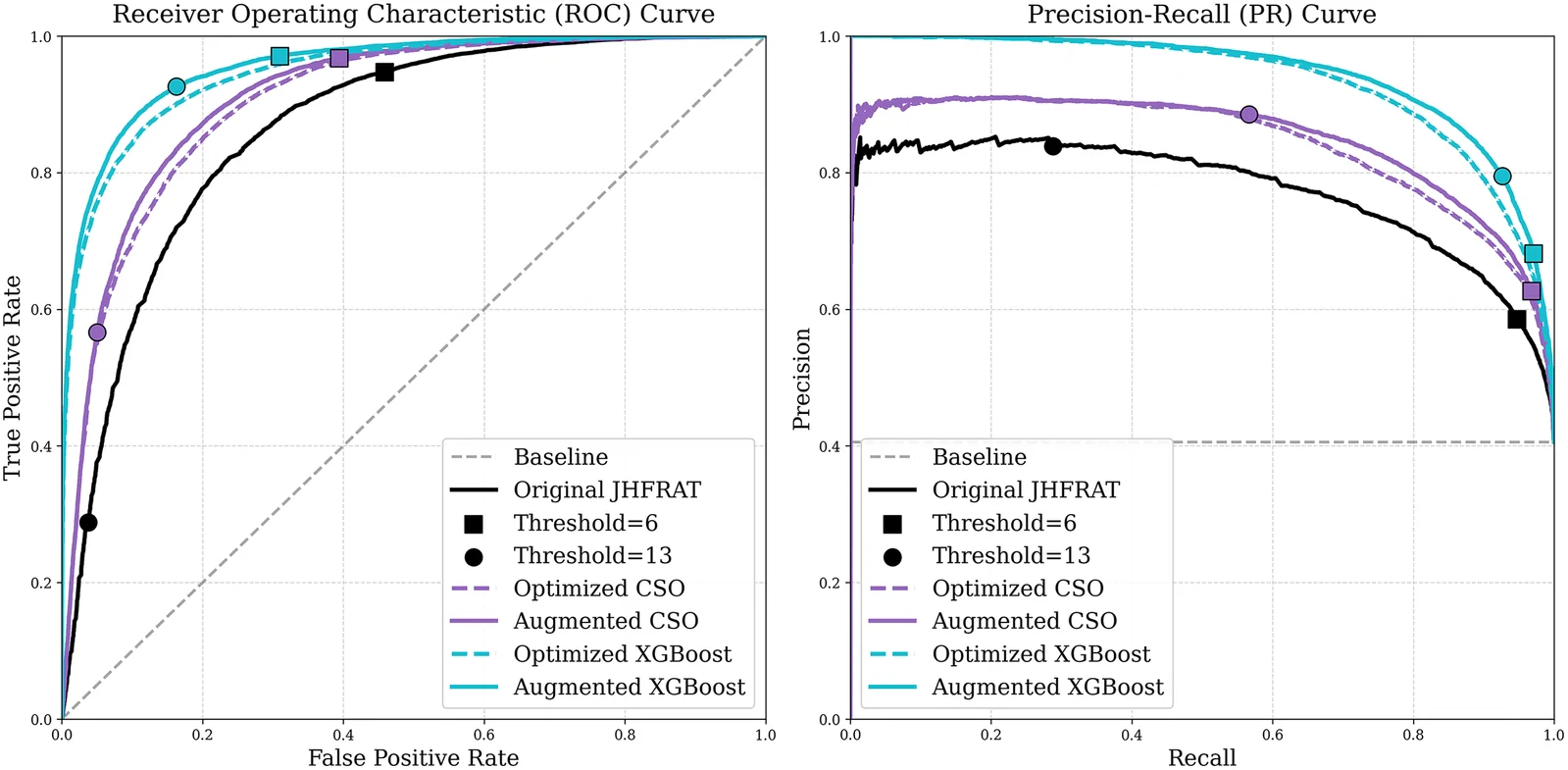
Receiver Operator Characteristic (ROC) and Precision-Recall (PR) curves for each model. All models improve on JHFRAT, with augmented models (with EHR variables) marginally improving on the models without EHR variables.

Analysis of model performance on the main study cohort demonstrates substantial improvements in fall risk assessment through optimization, with modest additional improvement from systematic integration of EHR variables. Table 2 provides key performance metrics for all models and Fig 4 shows the receiver operator characteristic curves and precision-recall curves, averaged across cross-validation folds, for each model. Across all recorded metrics the augmented CSO and augmented XGBoost perform similarly or slightly outperform their optimized (without EHR variables) counterparts and considerably outperform the current JHFRAT with the exception of specificity at the threshold of 13. These gains reflect score recalibration and improved separation of low- vs high-risk labels under the existing JHFRAT category thresholds, compared to post-hoc probability calibration. The largest performance metric gains are in the sensitivity at the threshold of 13, with the augmented CSO and XGBoost models achieving 0.57 and 0.92 sensitivity compared to the original JHFRAT’s sensitivity of 0.29. The decreases in specificity at the threshold of 13 are small enough, especially for CSO, that the subsequent CSO and XGBoost F1 scores also improve substantially from the original JHFRAT.

Table 3 displays the rates of classifying each type of patient (low-risk, high-risk, and indeterminate-risik label) for the original JHFRAT and each CSO model. Our augmented CSO model correctly reclassified 3,788 high-risk patients (27% of the high-risk cohort) from moderate- or low-risk categories. Simultaneously, the model identified 860 low-risk patients (4.2% of the low-risk cohort) who were previously over-classified. A modest tradeoff exists: 264 low-risk labeled patients (1.3%) were reclassified as high-risk.

**Table 3:** Number of patients per risk label and predicted risk category across models. Optimized and augmented CSO models increase the number of correctly classified patients and decrease the number of patients in the study cohort classified as moderate-risk. Bolded numbers constitute correctly classified encounters.

Intervention-Based Proxy Risk Label	Model	Patients in Predicted Risk Category (percent for risk label)		
		Low	Moderate	High
**Non-Fall Encounters**				
Low-Risk	Original	**10,953 *(54%)***	8,538 *(42%)*	670 *(3%)*
	Optimized CSO	**12,302 *(61%)***	6,905 *(34%)*	954 *(5%)*
	Augmented CSO	**12,254 *(61%)***	6,906 *(34%)*	1,001 *(5%)*
High-Risk	Original	725 *(5%)*	9,101 *(67%)*	**3,792 *(28%)***
	Optimized CSO	516 *(4%)*	5,741 *(42%)*	**7,361 *(54%)***
	Augmented CSO	444 *(3%)*	5,501 *(40%)*	**7,673 *(56%)***
Indeterminate-Risk	Original	3,340 *(26%)*	8,501 *(66%)*	985 (8%)
	Optimized CSO	3,405 *(27%)*	7,558 *(59%)*	1,863 *(15%)*
	Augmented CSO	3,199 *(25%)*	7,641 *(60%)*	1,986 *(15%)*
**Fall Encounters**				
Low-Risk	Original	***20 (19%)***	75 *(69%)*	13 *(12%)*
	Optimized CSO	**30 *(28%)***	64 *(59%)*	14 *(13%)*
	Augmented CSO	**25 *(23%)***	68 *(63%)*	15 *(14%)*
High-Risk	Original	7 *(3%)*	106 *(48%)*	**106 *(48%)***
	Optimized CSO	1 *(1%)*	60 *(27%)*	**158 *(72%)***
	Augmented CSO	3 *(1%)*	54 *(25%)*	**162 *(74%)***
Indeterminate-Risk	Original	3 *(3%)*	79 *(72%)*	28 *(26%)*
	Optimized CSO	3 *(3%)*	69 *(63%)*	38 *(35%)*
	Augmented CSO	3 *(3%)*	71 *(65%)*	36 *(33%)*

### 3.3. Feature contribution

Table 4 highlights the variable coefficients and their relative contribution between the baseline JHFRAT and each of the machine learning models. The relative feature importances for the JHFRAT variables do not vary significantly between the optimized and augmented models. Original JHFRAT components, such as cognition and mobility, retain substantial coefficients in the CSO models, reflecting their relevance to risk perception and intervention implementation. The marginal improvement gain from the addition of EHR variables also suggests that the existing JHFRAT items capture most of the relevant risk perception-driving clinical context. However, some variables, such as ‘One fall within 6 months before admission’, do not appear as significant in CSO models. The uniformly scored JHFRAT mobility items, appear with notably differing coefficients in CSO, and cognition items present ordinally different feature importances in CSO relative to JHFRAT. Notably, the percentage contributions of age, fall history, and medication are reduced in CSO compared with JHFRAT, while cognition, patient care equipment, and mobility have increased. Within the additional EHR variables, only AM-PAC and the service categories of neurosurgery, orthopedics, and neurology show notable contributions. Other variables, including gender and race, do not stand out. Tree SHAP analysis provides insights into the relative importance of individual features within the XGBoost framework. Among these, the AM-PAC mobility score and the JHFRAT 'requires assistance' mobility item emerge as the most influential features, maintaining consistency with their importance in the augmented CSO model.

**Table 4:** Comparison of variable contribution across models. The values for the items of the JHFRAT assessment are displayed as: coefficient (percentage of sum of coefficients for JHFRAT items only) to fairly compare feature importance. The occurrence rate, augmented CSO coefficients, and augmented XGBoost SHAP values are all reported as the average across cross-validation folds. Arrows indicate the change in contribution percentage compared to the JHFRAT baseline.

Feature Category	Variable	Encounters Average Occurrence by Risk Label			Baseline: JHFRAT Average Coefficient	Optimized CSO Average Coefficient	Augmented CSO Average Coefficient	Optimized XGBoost Average SHAP	Augmented XGBoost Average SHAP
		Excluded	Indeterminate	Low and High					
***JHFRAT Variables***									
***Age***	60 - 69 years	0.230	0.226	0.222	1 *(2.0%)*	0.6 *(1.2%)* ↓	0.3 *(0.6%)* ↓	-0.004	-0.031
	70 - 79 years	0.210	0.208	0.204	2 *(4.1%)*	1.0 *(2.0%)* ↓	0.7 *(1.3%)* ↓	-0.011	
	Greater than or equal to 80 years	0.142	0.107	0.137	3 *(6.1%)*	1.4 *(2.7%)* ↓↓	1.1 *(1.8%)* ↓↓	-0.006	
***Elimination, Bowel and Urine***	Incontinence	0.241	0.073	0.188	2 *(4.1%)*	3.1 *(5.9%)* ↑	2.4 *(4.0%)* ↑	-0.085	-0.094
	Urgency or frequency	0.085	0.080	0.078	2 *(4.1%)*	3.2 *(6.3%)* ↑	3.5 *(5.9%)* ↑↑	-0.004	-0.008
***Cognition***	Altered awareness of immediate physical environment	0.133	0.025	0.100	1 *(2.0%)*	4.1 *(7.9%)* ↑↑	3.6 *(6.1%)* ↑↑	0.019	0.024
	Impulsive	0.041	0.012	0.049	2 *(4.1%)*	9.1 *(17.5%)* ↑↑	8.4 *(14.1%)* ↑↑	0.043	0.038
	Lack of understanding of one’s physical and cognitive limitations	0.069	0.012	0.031	4 *(8.2%)*	1.0 *(1.9%)*	1.1 *(1.8%)* ↑	0.029	0.024
***Patient Care Equipment***	One present	0.390	0.452	0.415	1 *(2.0%)*	1.8 *(3.5%)* ↑	1.2 *(2.0%)* ↑	-0.011	-0.004
	Two present	0.224	0.211	0.225	2 *(4.1%)*	2.8 *(5.4%)* ↑	2.2 *(3.6%)* ↑	-0.027	-0.030
	Three or more present	0.168	0.104	0.137	3 *(6.1%)*	3.2 *(6.1%)*	3.1 *(5.1%)* ↑	-0.002	-0.003
***Fall History***	One fall within 6 months before admission	0.155	0.096	0.123	5 *(10.2%)*	0.0 *(0.0%)* ↓↓	0.0 *(0.0%)* ↓↓	0.005	0.007
***Medications***	On 1 high fall risk drug	0.328	0.349	0.348	3 *(6.1%)*	2.0 *(3.8%)* ↓	0.8 *(1.3%)* ↓↓	-0.018	-0.009
	On 2 or more high fall risk drugs	0.442	0.428	0.434	5 *(10.2%)*	2.3 *(4.5%)* ↓↓	1.2 *(2.0%)* ↓↓	-0.012	-0.016
	Sedated procedure within past 24 hours	0.043	0.033	0.031	7 *(14.3%)*	2.3 *(4.5%)* ↓↓	1.3 *(2.2%)* ↓↓	0.002	-0.002
***Mobility***	Requires assistance	0.567	0.491	0.549	2 *(4.1%)*	6.5 *(12.6%)* ↑↑	4.3 *(7.2%)* ↑↑	-0.259	-0.126
	Unsteady gait	0.092	0.057	0.082	2 *(4.1%)*	3.7 *(7.1%)* ↑	1.9 *(3.1%)* ↑	-0.017	-0.017
	Visual or auditory impairment affecting mobility	0.021	0.007	0.016	2 *(4.1%)*	3.7 *(7.1%)* ↑	2.0 *(3.4%)* ↓	0.003	0.002
*JHFRAT Variables Coefficient Sum Subtotal*					49 *(100%)*	52 *(100%)*	39.0 *(100%)*	N/A	N/A
***Additional EHR Variables***									
***AMPAC***	< 25	0.148	0.033	0.097			3.0		-0.162
	25-35	0.110	0.061	0.101			4.2		
	35-45	0.266	0.304	0.283			3.1		
	>45	0.336	0.465	0.393			0		
***JHHLM***	1-3	0.147	0.113	0.190			0		-0.010
	4-5	0.081	0.065	0.086			0.2		
	6-8	0.679	0.721	0.636			0.4		
***Documented Comorbidities***	<5	0.691	0.814	0.748			0		-0.001
	5-10	0.291	0.184	0.248			0.5		
	>10	0.018	0.002	0.004			0.3		
***Sex***	Female	0.496	0.511	0.501			0		0.000
	Male	0.503	0.489	0.499			0		0.000
***Race***	Black	0.322	0.320	0.335			0		0.000
	White	0.577	0.556	0.551			0		0.000
	Other	0.101	0.114	0.113			0.2		0.000
***Service Category***	Medicine	0.518	0.543	0.583			1.3		-0.007
	Surgery	0.167	0.207	0.180			0		0.007
	Oncology/Hematology	0.077	0.085	0.069			0.4		0.000
	Neurosurgery	0.076	0.057	0.057			2.8		-0.010
	Orthopedics	0.036	0.044	0.038			1.6		-0.006
	Neurology	0.050	0.020	0.032			2.5		-0.001
	Other	0.054	0.044	0.042			0		0.004
*Additional EHR Variables Coefficient Sum Subtotal*					0	0	20.5	N/A	N/A
*All Variables Coefficient Sum Total*					49	52	59.56	N/A	N/A

### 3.4. Differential risk scoring patterns

**Fig 5:**
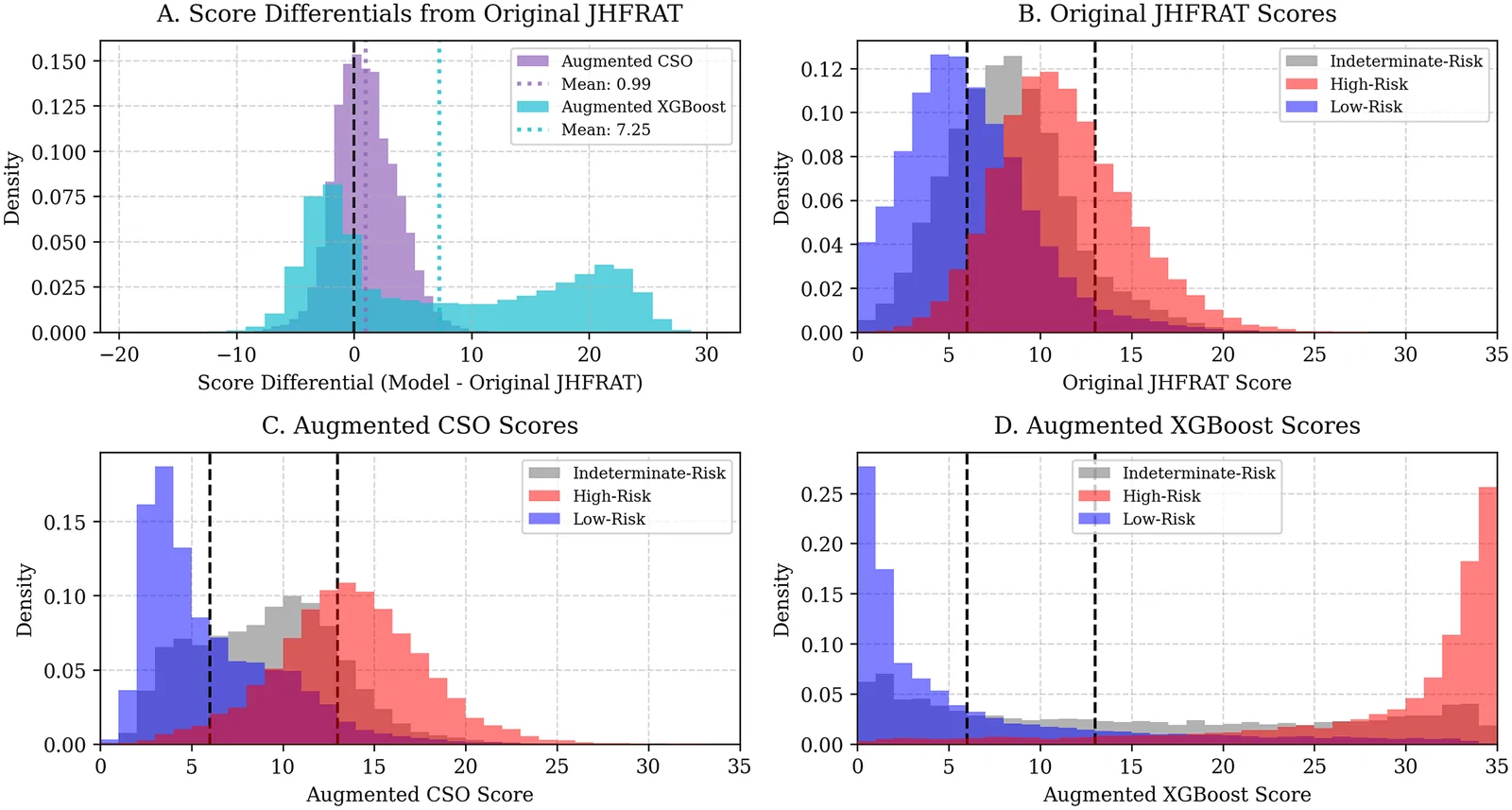
For all data in the study cohort, with the main cohort intervention-based proxy labels: A) Score differentials (model score – JHFRAT score) between the two augmented models and JHFRAT, B-C) Total risk score distributions per class for the original JHFRAT and the augmented CSO, respectively, for λ=0.5. D) Transformed XGBoost risk score, derived from multiplying output probability of high-risk by 35 (maximum possible original JHFRAT score).

Analysis of risk score differentials between the augmented and baseline JHFRAT frameworks demonstrates structured modification of risk assessment patterns. Fig 5 shows how the CSO differential distribution exhibit approximately normal characteristics with slight positive asymmetry, suggesting stable augmentation of baseline risk scores without introducing substantial variability. In contrast, the projected XGBoost scores vary more broadly from the original JHFRAT scores. Both the CSO and XGBoost models result in a reduction in the number of encounters in the study cohort considered moderate risk. For XGBoost, this is a direct result of the projected scores following the same skewness of the raw probabilities output, which favor values close to 0 and 1, rather than intermediate values, as seen in Fig 5D). The CSO score distributions more closely resemble those of the original JHFRAT, with a larger shift in the distribution of scores for the high-risk labeled encounters. This aligns with the augmented CSO results from Table 3, with a higher portion of high-risk labeled encounters moving from moderate risk to high risk (27.2 percentage points) than the portion of low-risk labeled encounters moving from moderate to low risk (4.2 percentage points).

### 3.5. Sensitivity analysis

**Fig 6:**
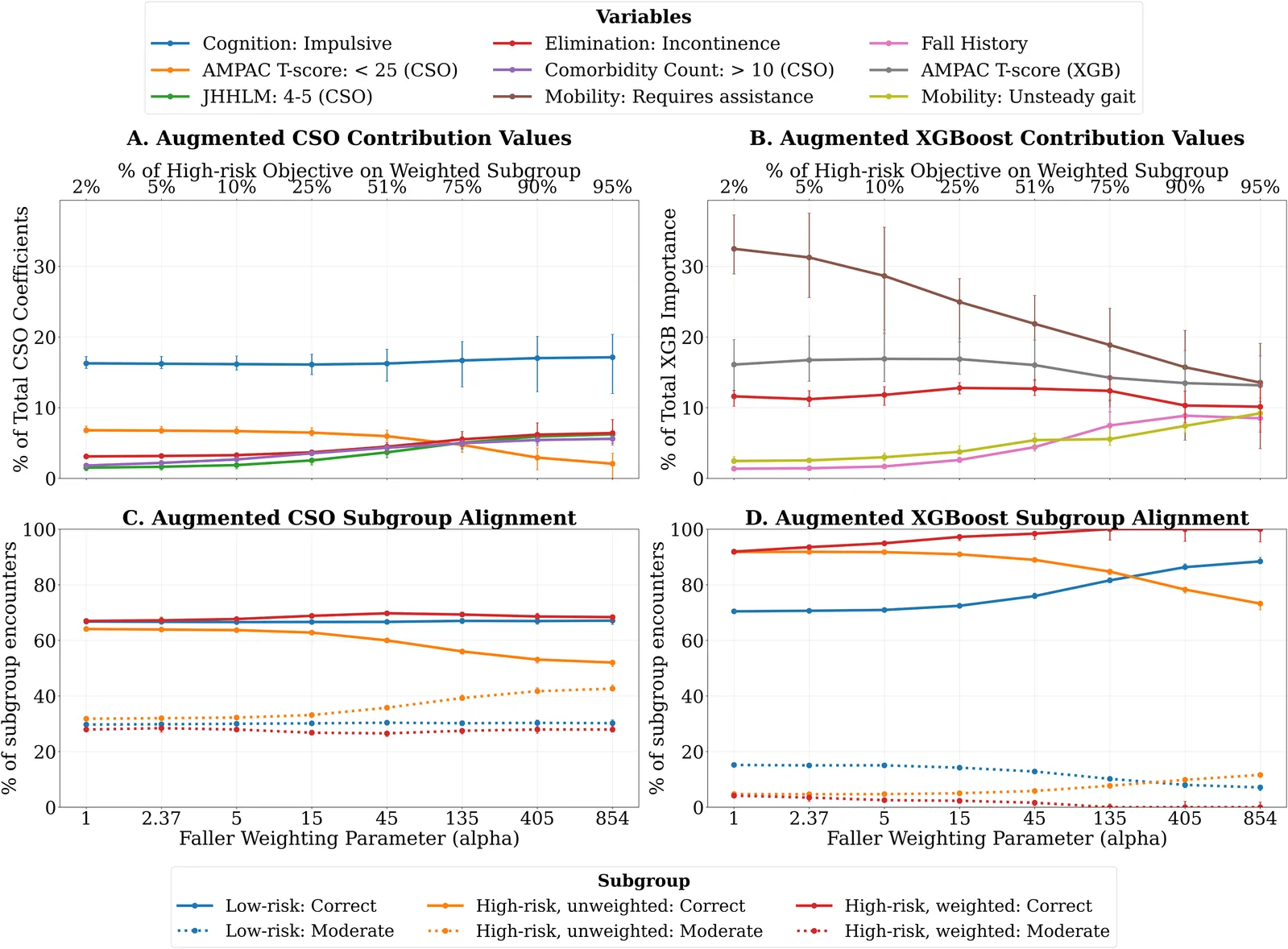
Fall occurrence high-risk override sensitivity analysis results for CSO (A and C) and XGBoost (B and D). A-B) Feature contribution metrics for the top 5 most variable coefficients across weighting parameter values and cross-validation folds. Error bars indicate maximum and minimum values across five validation folds for the given weighting parameter value. C-D) Classification alignment with intervention-based labels for low-risk (blue), high-risk non-fallers (orange), and high-risk fallers (red) across weighting parameter values, averaged across cross-validation folds, with error bars indicating maximum and minimum values across folds.

The feature importance results for the falls-based label override sensitivity analysis are displayed in Table 5 and Fig 6. The equivalent results for the high-intervention threshold and match-based label override sensitivity analyses are included in S1 Table, S1 Fig, and S2 Fig.

**Table 5:** Feature importance measures for the top five (per augmented model) highest % importance variance risk factors across the falls-based sensitivity analysis runs. Values for these top features are compared across both models, regardless of variance in the other model. For CSO, feature importance is calculated as the coefficient divided by the sum of all coefficients. For XGBoost, feature importance is calculated as the absolute value of the SHAP divided by the sum of all absolute SHAP values. Feature importance measures are calculated across all cross-validation folds across all hyperparameter α values.

Risk Factor	Model (Variance Rank)	Feature Importance (%) Measures			
		Variance	Median	Minimum	Maximum
**Augmented CSO Top 5 High-Variance Risk Factor Importances for Falls-based Override Sensitivity Analysis**					
AMPAC <= 25	CSO (1)	1.95	6.25	0.00	7.15
*AMPAC*	*XGBoost (3)*	*2.91*	*15.55*	*9.71*	*20.20*
JHHLM 4–5	CSO (2)	1.90	3.10	0.97	6.95
*JHHLM*	*XGBoost (20)*	*0.44*	*0.81*	*0.57*	*1.94*
Comorbidity Count > 10	CSO (3)	1.63	3.46	1.13	6.71
*Comorbidity Count*	*XGBoost (8)*	*0.81*	*1.18*	*0.69*	*3.30*
Impulsive (Cognition)	CSO (4)	1.40	16.29	14.70	20.00
	*XGBoost (13)*	*0.64*	*3.01*	*1.16*	*3.90*
Three or more Patient Care Equipment	CSO (5)	1.30	2.35	0.00	3.72
	*XGBoost (30)*	*0.12*	*1.06*	*0.73*	*1.25*
**Augmented XGBoost Top 5 High-Variance Risk Factor Importance for Falls-based Override Sensitivity Analysis**					
Requires Assistance (Mobility)	XGBoost (1)	7.51	23.58	6.51	34.24
	*CSO (16)*	*0.76*	*5.29*	*3.27*	*5.98*
Recent Fall History	XGBoost (2)	3.14	3.39	1.18	10.83
	*CSO (9)*	*1.13*	*1.96*	*1.03*	*4.65*
AMPAC	XGBoost (3)	2.91	15.55	9.71	20.20
*AMPAC <= 25*	*CSO (1)*	*1.95*	*6.25*	*0.00*	*7.15*
*AMPAC 25–35*	*CSO (14)*	*0.83*	*6.70*	*5.32*	*8.85*
*AMPAC 35–45*	*CSO (21)*	*0.57*	*4.52*	*3.99*	*6.33*
*AMPAC > 45*	*CSO (36)*	*0.00*	*0.00*	*0.00*	*0.00*
Unsteady Gait (Mobility)	XGBoost (4)	2.64	4.48	2.03	15.13
	*CSO (10)*	*1.11*	*5.15*	*3.95*	*8.37*
Incontinence (Elimination)	XGBoost (5)	1.72	11.49	7.72	15.78
	*CSO (6)*	*1.30*	*4.05*	*2.98*	*6.75*

The constrained score optimization feature importances remain stable as the weighting of fall encounters increases, while the XGBoost feature importances vary more substantially. The feature with the largest overall variation for CSO, comorbidity count, varies only by 4.39 percentage points, increasing from 0.81% to 5.2% of the total coefficient sum as α increases from 1 to 854 (corresponding to fall encounters increasing from 2% to 95% of the high-risk portion of the objective). The feature with the highest inter-fold variability is ‘impulsive’ in the cognition category, which is also the highest contributing feature overall. The two features with the largest importance variation for the XGBoost model are the two with the highest importance overall: ‘requires assistance’ in the mobility category (14.00 – 25.81% of absolute value SHAP sum), and the AM-PAC score (10.60 – 17.80% of absolute value SHAP sum). The average XGBoost 'requires assistance' feature importance decreases as α increases, while the AM-PAC feature importance neither increases nor decreases monotonically with increasing α. Both parameters have notable inter-fold variability, as indicated by the error bars in Fig 6.

The high-intensity threshold sensitivity and matching-based analyses reveals similar patterns of smaller feature importance variability in CSO compared to XGBoost. In both, ‘impulsive’ is the most variable CSO feature, and ‘requires assistance’ is the most variable XGBoost feature.

## 4. Discussion

### 4.1. Clinical impact and patient safety outcomes

Data-driven optimization of the JHFRAT meaningfully improves fall risk stratification with immediate implications for patient safety and workflow efficiency. In practical terms, the augmented CSO model performance translates to a weekly average of an additional 35 Johns Hopkins Health System patients who are perceived as high risk (per our proxy labels) being classified by JHFRAT as high risk, while only 2–3 low-risk patients per week would be classified as high-risk despite being perceived low-risk (per the proxy labels). Concurrently, approximately 8 additional perceived low-risk patients would be labeled as low-risk. Sensitivity analysis reveals that CSO coefficients change minimally when the model is trained to prioritize categorizing fall encounters (regardless of interventions) as high-risk. This stability indicates that clinician-perceived fall risk, as operationalized through intervention patterns, encodes similar underlying risk factor patterns to those associated with actual fall occurrences, providing external support for the validity of this operationalization. Further analysis is required to better understand the unmediated causal relationships between the risk factors and falls. Nevertheless, the improved score alignment with intervention-based labels of clinician-perceived risk is an important first step in standardizing and improving the JHFRAT such that it better reflects clinical judgment of fall risk.

### 4.2. Interpretation of key findings and contributions

The central contribution of this study is a principled framework for recalibrating a bedside fall risk assessment tool to better align with clinician-perceived fall risk, operationalized through systematically observed intervention patterns. This framing sidesteps the limitations of outcome-based optimization, which is constrained by infrequent fall events and the confounding effects of the very interventions being evaluated. Instead, we target the clinical judgment embedded in nurses’ preventive care decisions. The coherence of the resulting model reflects the success of this approach: recalibrated JHFRAT coefficients retain clinical interpretability, align with established fall risk literature, and remain stable across sensitivity analyses that use alternative labeling strategies, including one that overrides all fall encounters as high-risk.

The recalibrated score distributions illustrate how the optimization refines, rather than disrupts, the existing JHFRAT framework. Average score differentials of 0.94 and 1.01 points for the optimized and augmented CSO models, respectively, indicate meaningful re-weighting of risk factors without score inflation, preserving the diagnostic utility of the original scale. The dual-threshold structure distinguishing low-risk, indeterminate-risk, and high-risk categories is retained by design, ensuring that clinicians can continue applying established care protocols without retraining or workflow disruption. Together, these properties make CSO recalibration a practically deployable path for evidence-based JHFRAT improvement. A secondary finding is that supplementing the 18 JHFRAT items with EHR-derived variables, particularly AM-PAC mobility scores and medical service category, yields modest additional discriminative performance. This suggests that some factors outside of the JHFRAT risk items may moderately influence clinical decisions on the application of prevention interventions. That demographic variables such as sex and race contribute minimally across all models is also noteworthy, indicating that the clinician-perceived fall risk signal captured here does not depend meaningfully on these factors on average.

For context, we additionally trained an XGBoost model as a performance benchmark. While XGBoost achieves higher ROC metrics, its outputs are not directly usable in a clinical setting that relies on the JHFRAT score for care decisions. Without score clipping, projected XGBoost risk scores distribute to unrealistic extremes on the JHFRAT scale and lose interpretive meaning. The CSO approach trades some statistical performance for a recalibrated score that retains meaning on the familiar clinical scale, is auditable by clinical staff, and integrates into existing nursing workflows without modification. This tradeoff reflects the broader principle that the appropriate benchmark for a clinical decision-support tool is fit for the operational context in which it will be used, not raw discriminative performance alone [22–24].

### 4.3. Limitations and future directions

Several limitations warrant consideration for future implementation and research. Our retrospective design and intervention-based risk labeling approach reflects clinically-perceived risk through preventive care patterns rather than intrinsic biologic susceptibility and introduces confounding by indication. By design, the JHFRAT scores influence intervention decisions, and therefore the intervention-based labels introduce endogeneity from which our models benefit. Application of our models to predicting fall events without confounding of interventions may yield smaller performance gains.

A few key limitations may limit generalizability and deployment realism. Though the study data spans three hospitals, all hospitals are within the same health system. The use of AM-PAC and JH-HLM mobility assessments limits immediate generalizability to institutions without these standardized mobility measures, though alternative mobility indicators routinely collected in EHRs (such as physical therapy assessments or ambulation orders) could potentially substitute for these variables with appropriate validation. The systematic exclusion of encounters based on JHFRAT data availability and length of stay introduces biases to the study cohort by reducing patient heterogeneity in the model training and validation, potentially inflating model performance. Encounters with very brief or prolonged admissions are clinically important cases, albeit outside of the majority, which may require focused analysis for tailored fall risk assessment not addressed in this study. Importantly, the aggregation of risk factor values over the encounter data samples does not reflect the clinical reality of twice-daily JHFRAT use. Future retrospective analysis can deploy a thorough consideration of change in risk factors over time, such that the optimized model coefficients reflect moment-in-time risk while ignoring clinically insignificant transient change in the patient’s risk. Future prospective studies could validate the study findings in controlled settings and explore real-time risk prediction models that continuously update patient risk scores based on changing clinical status throughout hospitalization. Such models could provide even more precise risk stratification and intervention timing recommendations.

Future validation studies should examine the practical feasibility of translating these optimized coefficients into clinical practice, including the technical challenges of EHR integration and the organizational factors that influence adoption of modified risk assessment tools. Additionally, prospective evaluation of clinical outcomes following implementation would be essential to confirm the predicted improvements in fall prevention and resource allocation observed in our retrospective analysis. Use of sensor data, including from wearables, as a contributing factor in the risk assessment could help provide additional predictive power, as such data has been shown valuable for fall prediction in both outpatient and inpatient settings [25–27].

## Supporting information

S1 Table Feature importance measures for the top five (per augmented model) highest % importance variance risk factors across each of the sensitivity analysis runs. Values for these top features are compared across both models, regardless of variance in the other model. For CSO, feature importance is calculated as the coefficient divided by the sum of all coefficients. For XGBoost, feature importance is calculated as the absolute value of the SHAP divided by the sum of all absolute SHAP values. Feature importance measures are calculated across all cross-validation folds across all hyperparameter α values.
(DOCX)

S1 Fig High-intervention threshold sensitivity analysis results for CSO (A) and XGBoost (B). Feature contribution metrics for the top 5 most variable coefficients across weighting parameter values and cross-validation folds. Error bars indicate maximum and minimum values across five validation folds for the given weighting parameter value.
(TIFF)

S2 Fig Prior-to-falls intervention pattern matching sensitivity analysis results for CSO (A and C) and XGBoost (B and D). A-B) Feature contribution metrics for the top 5 most variable coefficients across weighting parameter values and cross-validation folds. Error bars indicate maximum and minimum values across five validation folds for the given weighting parameter value. C-D) Classification alignment with intervention-based labels for low-risk (blue), high-risk non-fallers (orange), and high-risk fallers (red) across weighting parameter values, averaged across cross-validation folds.
(TIFF)
