# Supplementary material for: An interpretable data-driven approach to optimizing clinical fall risk assessment
Source: PLOS Digit Health. 2026 Sep 1;5(9):e0001631. doi: 10.1371/journal.pdig.0001631 (PMC13533424; doi:10.1371/journal.pdig.0001631)
Supplement: Supplementary file 1 [file pdig.0001631.s001.docx]

| **Risk Factor** | **Model (Variance Rank)** | **Feature Importance (%) Measures** | | | |
| --- | --- | --- | --- | --- | --- |
|  |  | **Variance** | **Median** | **Minimum** | **Maximum** |
| **Augmented CSO Top 5 High-Variance Risk Factor Importances for Falls-based Override Sensitivity Analysis** | | | | | |
| AMPAC <= 25 | CSO (1) | 1.95 | 6.25 | 0.00 | 7.15 |
| *AMPAC* | *XGBoost (3)* | *2.91* | *15.55* | *9.71* | *20.20* |
| JHHLM 4-5 | CSO (2) | 1.90 | 3.10 | 0.97 | 6.95 |
| *JHHLM* | *XGBoost (20)* | *0.44* | *0.81* | *0.57* | *1.94* |
| Comorbidity Count > 10 | CSO (3) | 1.63 | 3.46 | 1.13 | 6.71 |
| *Comorbidity Count* | *XGBoost (8)* | *0.81* | *1.18* | *0.69* | *3.30* |
| Impulsive (Cognition) | CSO (4) | 1.40 | 16.29 | 14.70 | 20.00 |
|  | *XGBoost (13)* | *0.64* | *3.01* | *1.16* | *3.90* |
| Three or more Patient Care Equipment | CSO (5) | 1.30 | 2.35 | 0.00 | 3.72 |
|  | *XGBoost (30)* | *0.12* | *1.06* | *0.73* | *1.25* |
| **Augmented XGBoost Top 5 High-Variance Risk Factor Importance for Falls-based Override Sensitivity Analysis** | | | | | |
| Requires Assistance (Mobility) | XGBoost (1) | 7.51 | 23.58 | 6.51 | 34.24 |
|  | *CSO (16)* | *0.76* | *5.29* | *3.27* | *5.98* |
| Recent Fall History | XGBoost (2) | 3.14 | 3.39 | 1.18 | 10.83 |
|  | *CSO (9)* | *1.13* | *1.96* | *1.03* | *4.65* |
| AMPAC | XGBoost (3) | 2.91 | 15.55 | 9.71 | 20.20 |
| *AMPAC <= 25* | *CSO (1)* | *1.95* | *6.25* | *0.00* | *7.15* |
| *AMPAC 25-35* | *CSO (14)* | *0.83* | *6.70* | *5.32* | *8.85* |
| *AMPAC 35-45* | *CSO (21)* | *0.57* | *4.52* | *3.99* | *6.33* |
| *AMPAC > 45* | *CSO (36)* | *0.00* | *0.00* | *0.00* | *0.00* |
| Unsteady Gait (Mobility) | XGBoost (4) | 2.64 | 4.48 | 2.03 | 15.13 |
|  | *CSO (10)* | *1.11* | *5.15* | *3.95* | *8.37* |
| Incontinence (Elimination) | XGBoost (5) | 1.72 | 11.49 | 7.72 | 15.78 |
|  | *CSO (6)* | *1.30* | *4.05* | *2.98* | *6.75* |
| **Augmented CSO Top 5 High-Variance Risk Factor Importances for High-Intervention Threshold Sensitivity Analysis** | | | | | |
| Impulsive (Cognition) | CSO (1) | 1.17 | 15.59 | 13.76 | 19.01 |
|  | *XGBoost (5)* | *0.54* | *3.23* | *2.24* | *4.40* |
| Altered Awareness (Cognition) | CSO (2) | 1.00 | 8.66 | 7.16 | 10.87 |
|  | *XGBoost (4)* | *0.78* | *5.05* | *3.71* | *6.22* |
| Visual/Auditory Impairment (Mobility) | CSO (3) | 0.64 | 3.18 | 1.76 | 4.19 |
|  | *XGBoost (14)* | *0.21* | *0.85* | *0.61* | *1.45* |
| JHHLM 6-8 | CSO (4) | 0.57 | 0.97 | 0.13 | 1.76 |
| *JHHLM* | *XGBoost (30)* | *0.05* | *0.60* | *0.55* | *0.70* |
| JHHLM 4-5 | CSO (5) | 0.49 | 1.44 | 0.61 | 2.20 |
| *JHHLM* | *XGBoost (30)* | *0.05* | *0.60* | *0.55* | *0.70* |
| **Augmented XGBoost Top 5 High-Variance Risk Factor Importances for High-Intervention Threshold Sensitivity Analysis** | | | | | |
| Requires Assistance (Mobility) | XGBoost (1) | 9.86 | 29.64 | 17.40 | 44.80 |
|  | *CSO (13)* | *0.24* | *5.94* | *5.58* | *6.51* |
| AMPAC | XGBoost (2) | 7.03 | 17.38 | 6.87 | 27.98 |
| *AMPAC <= 25* | *CSO (10)* | *0.29* | *6.77* | *6.11* | *7.48* |
| *AMPAC 25-35* | *CSO (8)* | *0.37* | *7.01* | *6.38* | *7.85* |
| *AMPAC 35-45* | *CSO (12)* | *0.27* | *4.28* | *3.72* | *4.67* |
| *AMPAC > 45* | *CSO (27)* | *0.11* | *0.00* | *0.00* | *0.36* |
| Incontinence (Elimination) | XGBoost (3) | 1.17 | 10.76 | 9.55 | 13.57 |
|  | *CSO (11)* | *0.27* | *3.09* | *2.62* | *3.69* |
| Altered Awareness (Cognition) | XGBoost (4) | 0.78 | 5.05 | 3.71 | 6.22 |
|  | *CSO (2)* | *1.00* | *8.66* | *7.16* | *10.87* |
| Impulsive (Cognition) | XGBoost (5) | 0.54 | 3.23 | 2.24 | 4.40 |
|  | *CSO (1)* | *1.17* | *15.59* | *13.76* | *19.01* |
| **Augmented CSO Top 5 High-Variance Risk Factor Importances for Matching-based Override Sensitivity Analysis** | | | | | |
| AMPAC <= 25 | CSO (1) | 2.43 | 5.83 | 0.00 | 7.36 |
| *AMPAC* | *XGBoost (2)* | *3.79* | *19.41* | *13.12* | *29.05* |
| Impulsive (Cognition) | CSO (2) | 2.08 | 16.95 | 14.74 | 23.11 |
|  | *XGBoost (6)* | *1.30* | *3.64* | *2.79* | *7.42* |
| Comorbidity Count > 10 | CSO (3) | 1.96 | 3.55 | 1.02 | 7.11 |
| *Comorbidity Count* | *XGBoost (14)* | *0.46* | *0.93* | *0.63* | *2.49* |
| Incontinence (Elimination) | CSO (4) | 1.50 | 4.03 | 2.99 | 7.72 |
|  | *XGBoost (3)* | *1.74* | *11.52* | *8.82* | *15.70* |
| Orthopedics (Service Category) | CSO (5) | 1.11 | 1.82 | 0.00 | 2.71 |
|  | *XGBoost (17)* | *0.35* | *1.30* | *0.55* | *2.39* |
| **Augmented XGBoost Top 5 High-Variance Risk Factor Importances for Matching-based Override Sensitivity Analysis** | | | | | |
| Requires Assistance (Mobility) | XGBoost (1) | 7.56 | 24.24 | 4.25 | 37.58 |
|  | *CSO (13)* | *0.84* | *5.17* | *3.39* | *6.26* |
| AMPAC | XGBoost (2) | 3.79 | 19.41 | 13.12 | 29.05 |
| *AMPAC <= 25* | *CSO (1)* | *2.43* | *5.83* | *0.00* | *7.36* |
| *AMPAC 25-35* | *CSO (16)* | *0.75* | *7.35* | *6.43* | *9.22* |
| *AMPAC 35-45* | *CSO (11)* | *0.94* | *4.85* | *3.95* | *6.99* |
| *AMPAC > 45* | *CSO (39)* | *0.00* | *0.00* | *0.00* | *0.00* |
| Incontinence (Elimination) | XGBoost (3) | 1.74 | 11.52 | 8.82 | 15.70 |
|  | *CSO (4)* | *1.50* | *4.03* | *2.99* | *7.72* |
| Unsteady Gait (Mobility) | XGBoost (4) | 1.67 | 3.71 | 1.90 | 8.23 |
|  | *CSO (17)* | *0.73* | *4.25* | *3.54* | *6.31* |
| Recent Fall History | XGBoost (5) | 1.31 | 2.01 | 1.08 | 5.78 |
|  | *CSO (15)* | *0.79* | *1.46* | *0.96* | *3.66* |

S1 Table: Feature importance measures for the top five (per augmented model) highest % importance variance risk factors across each of the sensitivity analysis runs. Values for these top features are compared across both models, regardless of variance in the other model. For CSO, feature importance is calculated as the coefficient divided by the sum of all coefficients. For XGBoost, feature importance is calculated as the absolute value of the SHAP divided by the sum of all absolute SHAP values. Feature importance measures are calculated across all cross-validation folds across all hyperparameter ⍺ values.
